# A High-Salt Diet Disturbs the Development and Function of Natural Killer Cells in Mice

**DOI:** 10.1155/2020/6687143

**Published:** 2020-12-21

**Authors:** Xiaokang Zeng, Yan Li, Weibiao Lv, Xinhuai Dong, Chong Zeng, Liming Zeng, Zibo Wei, Xu Lin, Yanning Ma, Qiang Xiao

**Affiliations:** ^1^Central Laboratory, Shunde Hospital, Southern Medical University (The First People's Hospital of Shunde Foshan), Foshan, 528300 Guangdong, China; ^2^Clinical Medicine Research Center, Shunde Hospital, Southern Medical University (The First People's Hospital of Shunde Foshan), Foshan, 528300 Guangdong, China; ^3^Clinical Laboratory, Shunde Hospital, Southern Medical University (The First People's Hospital of Shunde Foshan), Foshan, 528300 Guangdong, China; ^4^Radiotherapy Department, Shunde Hospital, Southern Medical University (The First People's Hospital of Shunde Foshan), Foshan, Guangdong, China; ^5^Pulmonary and Critical Care Medicine, Shunde Hospital, Southern Medical University (The First People's Hospital of Shunde Foshan), Foshan, 528300 Guangdong, China

## Abstract

A high-salt diet (HSD) is common worldwide and can lead to cardiovascular disease, chronic inflammation, and autoimmune diseases. Moreover, increasing evidence shows that HSD is closely related to a variety of immune diseases. Natural killer (NK) cells are important innate immune cells that directly kill their targets via degranulation and secretion of interferon gamma (IFN-*γ*). NK cells play a vital role in resisting viruses and preventing the malignant transformation of cells; however, whether HSD affects the development and function of NK cells has not yet been elucidated. Therefore, the purpose of the present study was to understand the effects of HSD on the development and function of NK cells, in addition to investigating the underlying molecular mechanism. Our results show that the number of NK cells in the spleen and lungs of HSD-fed mice was significantly reduced, which may be due to the inhibition of NK cell proliferation. Further, the development of NK cells in mice was evaluated, and it was found that HSD reduced the effective NK cell subset (CD27^+^CD11b^−^). Moreover, it was also found that the ability of NK cells to secrete CD107a and IFN-*γ* in HSD-fed mice was decreased following stimulation with RMA-S and YAC-1 tumor cells. Finally, the underlying molecular mechanism was evaluated, and it was found that HSD increased the production of reactive oxygen species (ROS) by NK cells, while the expression of CD122 was decreased, suggesting that HSD downregulates CD122 expression in NK cells via ROS signaling, thereby reducing the responsiveness to IL-15 and ultimately inhibiting NK cell function. The present research discovered a novel mechanism by which HSD inhibits the function of NK cells, providing an alternative avenue for the treatment of immune diseases caused by HSD.

## 1. Introduction

Natural killer (NK) cells are a type of lymphocyte that develop from hematopoietic stem cells in the bone marrow. During development, NK cells continuously acquire functional receptors and gradually mature [1], eventually directly destroying pathogen-infected or transformed cells and playing an important role in innate immunity [2]. Upon stimulation by external stimuli, NK cells release perforin and granzymes to clear transformed cells; perforin forms pores in the membrane of target cells, through which granzymes enter to induce apoptosis [3, 4]. Moreover, several tumor necrosis factor family ligands have been found to be upregulated, in addition to increased secretion of a variety of cytokines [5, 6]. Although NK cells play a crucial role in innate immunity, their differentiation mechanisms remain poorly understood.

A high-salt diet (HSD) refers to a daily salt intake greater than 5 g per person, and both public health agencies and guidelines recommend reducing salt intake [7]. A long-term HSD can cause many disorders such as cardiovascular disease, cancer, chronic inflammation, and autoimmune diseases [8, 9], but mechanistic understanding is lacking. Most studies have focused on the role of HSD in the kidney and the sympathetic nervous system and their direct effects on the vasculature [10]. Furthermore, increasing evidence shows that HSD weakens the immune system and promotes the development of hypertension by stimulating proinflammatory T cells [11]. Recently, it was demonstrated that increasing salt uptake promotes the occurrence of autoimmune encephalomyelitis by improving the function of T helper 17 cells and promoting the polarization of proinflammatory macrophages, thereby aggravating the autoimmune response of the central immune system [12]. High-dose salt treatment promotes the differentiation of follicular T helper cells (Tfh), accelerating the development of lupus syndrome in mice [13]; however, whether HSD affects the development and function of NK cells remains unclear.

Hence, the goal of the present study was to explore whether HSD disturbs the development and function of NK cells. We found that HSD-fed mice had a slightly decreased percentage and absolute number of NK cells in the spleen and lungs. Although small changes in number do not affect the cytotoxic function of NK cells, the secretion of CD107a and interferon gamma (IFN-*γ*) was decreased following tumor stimulation of NK cells from HSD-fed mice. Subsequently, we evaluated NK cell development in HSD-fed mice and found that the percentage and absolute number of effective NK cells (CD27^+^CD11b^+^) were significantly reduced, which may be the mechanism underlying the weakening of NK cell function in HSD-fed mice. We further explored the molecular mechanism, demonstrating that the expression of the IL-15 receptor beta chain-CD122 was decreased, while the transcription factors, Eomes, T-bet, and E4BP4, were normal in HSD-fed mice. Interestingly, the level of reactive oxygen species (ROS) in NK cells was enhanced in HSD-fed mice and increased expression of NADPH-oxidate subunits, gp91phox and p47phox, which represent the major participants in ROS production. We postulate that HSD disturbs NK cell function via retardation of cell development and enhancement of oxidative stress.

## 2. Materials and Methods

### 2.1. Animals and Experimental Design

The protocols used for animal experiments were approved by the Institutional Animal Care and Use Committee of Southern Medical University. All procedures involving animals were approved by the Animal Ethics Committee of Southern Medical University. C57BL/6J male mice (8-week-old) were obtained from the Guangdong Medical Animal Laboratory (Foshan, China). The mice were housed in a temperature-controlled (22 ± 1°C) environment on a 12 h/12 h light-dark cycle and provided with water and food *ad libitum* as previously described [14]. The mice received normal chow and tap water (control group) or sodium-rich chow containing 4% NaCl (Guangdong Animal Experiment Center; catalog number 20190217) and tap water containing 1% NaCl (HSD) for two months [15].

### 2.2. Real-Time Quantitative PCR

Total RNA was extracted from sorted splenic NK cells, from which cDNA was synthesized using the reverse transcription system (Takara, RR047A, Tokyo, Japan). Quantitative real-time polymerase chain reaction (RT-qPCR) was performed on a Bio-Rad CFX96 qPCR system using a SYBR ExScript PCR Kit (Takara, RR820A, Tokyo, Japan). The primer sequences of the selected genes used in the present study are shown in Table 1. The relative expression levels were calculated by the 2^-*ΔΔ*CT^ method.

### 2.3. Flow Cytometry

A BD FACSCanto flow cytometry analyzer (BD Biosciences) was used to perform the experiments. For the detection of extracellular proteins, lymphocytes were isolated and resuspended in ice-cold PBS containing 2% FBS and stained with monoclonal antibodies against mouse CD3 (17A2), NK1.1 (PK136), Nkp46 (29A1.4), CD27 (LG.7F9), CD11b (M1/70), IFN-*γ* (XMG1.2), CD107a (1D4B), annexin V (JF50-11), CD25 (PC61.5), CD69 (H1.2F3), and CD122 (5H4). Isotype controls were purchased from eBioscience (San Diego, CA). The expression level is expressed as the mean fluorescence intensity (MFI) following subtraction of the isotype control. For the detection of intracellular proteins, such as Ki67 (SOLA15), E4BP4 (S2M-E19), Eomes (DAN11MAG), and T-bet (4B10) (eBioscience), lymphocytes were fixed with Phosflow Lyse/Fix buffer and permeabilized with Phosflow Perm buffer III (BD) prior to staining with antibodies (given in parentheses). The data were analyzed using FlowJo software.

### 2.4. Detection of the Intracellular Expression of CD107a and IFN-*γ*

Each mouse was injected with 200 *μ*g poly I:C (P9582, Sigma, MO, US) 18 h prior to harvest to prime the NK cells. The primed splenic NK cells (2 × 10^6^) were cocultured for 4 h with the same number of RMA-S (the murine RMA-S mutant cell lines have a defect in class I assembly and express markedly reduced levels of class I molecules at the cell surface) and YAC-1 (target of NK cells, murine T cell lymphoma, have defects in MHC class I expression) tumor cells. Monoclonal antibodies against CD107a and IFN-*γ* or the respective isotype controls were subsequently added. The GolgiStop™ reagent (554724, BD Biosciences, CA, US) was added to inhibit the secretion of intracellular CD107a and IFN-*γ*. Medium was used as the negative control. After stimulation, cells were harvested, washed with PBS, and subjected to FACS to detect the expression of IFN-*γ* and CD107a.

### 2.5. Detection of ROS by FACS

NK cells were sorted by FACS and seeded onto 24-well plates, followed by incubation with 1x ROS staining solution (MAK143, Sigma, MO, US) for 60 min at 37°C, 5% CO_2_. Subsequently, the NK cells were stimulated with 0.5 *μ*g/mL LPS for 24 h. The intensity of ROS was analyzed by flow cytometry.

### 2.6. Western Blotting

Splenic NK cells from control and HSD-fed mice were sorted by FACS. Total protein was extracted according to the manufacturer's instructions (P0033, Beyotime, Shanghai). Protein extracts were separated by SDS-PAGE on 12% polyacrylamide gels and transferred to a PVDF membrane (3010040001, Sigma, MO, US). After blocking with 5% (*w*/*v*) BSA at room temperature for 1 h, the membranes were incubated overnight at 4°C with primary antibody (anti-gp91phox, EPR6991; anti-p47phox, ab166930; and *β*-actin, ab8226) (Abcam, Cambridge, US). The following day, the membranes were washed in PBS and incubated for 1 h with an HRP-conjugated secondary antibody (Beyotime, Shanghai, China). Protein bands were detected using an enhanced chemiluminescence kit (Thermo Scientific, Hudson, NH, USA) according to the manufacturer's instructions.

### 2.7. Statistical Analysis

Unpaired Student's *t*-test (two-tailed) was performed using SPSS 22.0 (SPSS Inc., Chicago, IL, US). A *P* value < 0.05 was considered significant. ^∗^*P* < 0.05, ^∗∗^*P* < 0.01, and ^∗∗∗^*P* < 0.001. Data are shown as the mean ± SEM.

## 3. Results

### 3.1. The mRNA Levels of Activation and Functional Markers Are Downregulated in NK Cells following Culture in High-Salt Medium

**Figure 1:**
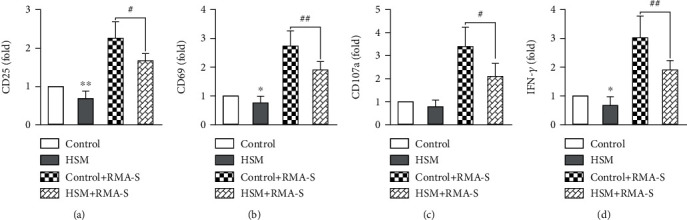
The genes encoding activation and functional markers of NK cells are downregulated following culture in high-salt medium. Mouse splenic NK cells were sorted by FACS and cultured in normal or high-salt medium with or without coculture with RMA-S. Quantitative reverse transcription-PCR (RT-qPCR) was used to analyze the mRNA levels of the activation genes, CD25 (a) and CD69 (b), and the functional genes, CD107a (c) and IFN-*γ* (d), in NK cells. Data represent at least three independent experiments and are shown as the mean ± SEM. Unpaired Student's *t*-test (two-tailed) was performed using the Prism software. A *P* value <0.05 was considered significant. ^∗^*P* < 0.05, ^∗∗^*P* < 0.01.

Firstly, we clarified the physiological effects of high-salt stimulation on NK cells. Mouse splenic NK cells were sorted by flow cytometry and cultured for 24 h in high-salt medium containing 4 mM NaCl, to which 1000 U/mL IL-2 was added to stimulate growth. The mRNA levels of the NK cell activation markers, CD25 and CD69, were measured by RT-qPCR. The data show that these levels were significantly decreased in NK cells from the high-salt group (Figures 1(a) and 1(b)), indicating that high salt may inhibit NK cell activation. We further tested the NK cell degranulation index, CD107a, and cell killing index, IFN-*γ*, and the results show that their mRNA expression levels in NK cells were significantly reduced following high-salt treatment (Figures 1(c) and 1(d)). These results indicate that high-salt treatment inhibits NK cell function. We speculate that mice subjected to long-term HSD will suffer damage to the immune surveillance function of NK cells.

### 3.2. HSD Decreases the Percentage and Absolute Number of NK Cells in the Spleen and Lungs

**Figure 2:**
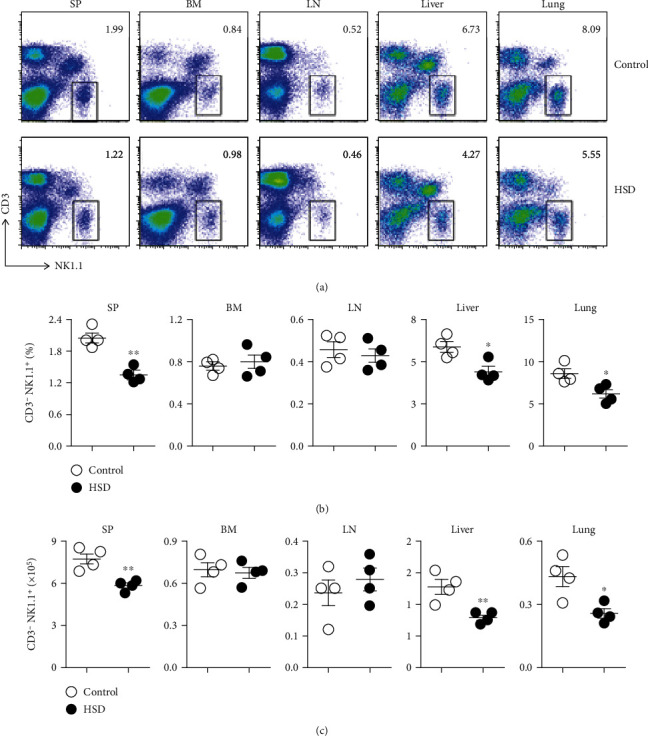
The number of NK cells in the spleen and lungs of HSD-fed mice was decreased. Representative flow cytometry profiles of CD3^−^ NK1.1^+^ NK cells from the spleen (SP), bone marrow (BM), lymph nodes (LN), liver, and lungs of control or HSD-fed mice (a). The percentage of NK cells in the indicated organs and tissues from control and HSD-fed mice (b). The absolute number of NK cells in the indicated organs and tissues from control and HSD-fed mice (c). Each symbol represents an individual mouse. All the data represent at least three independent experiments. Data are shown as the means ± SEM. Unpaired Student's *t*-tests (two-tailed) were performed using the Prism software. A *P* value of <0.05 was considered significant. ^∗^*P* < 0.05, ^∗∗^*P* < 0.01.

Maintaining a normal number of NK cells in the immune tissues plays an important role in antitumor immunity. Excessive intake of dietary salt (NaCl) is clearly defined as unhealthy due to its risk of inducing chronic inflammation, cardiovascular disease, and autoimmune diseases; hence, we wondered whether HSD-fed mice would have disturbed NK cell development and function. We first detected NK cells in different organs or tissues from control and HSD-fed mice. The data show that the percentage and absolute number of NK cells (CD3^−^ NK1.1^+^) [16] in the spleen and lungs of HSD-fed mice were reduced as compared with those of control mice (Figures 2(a)−2(c)). The percentage of NK cells in the spleen and lungs of HSD-fed mice was decreased by 34% and 41%, respectively, as compared with that in control mice (Figures 2(a) and 2(b)), and the absolute number of NK cells was decreased by 24% and 41%, respectively, as compared with that in control mice (Figure 2(c)). Hence, these data indicate that HSD-fed mice may have decreased NK cell-mediated immune surveillance.

### 3.3. HSD Decreases the Proportion of CD27^+^CD11b^+^ NK Cells in the Spleen and Inhibits NK Cell Maturation in the Spleen and Bone Marrow

**Figure 3:**
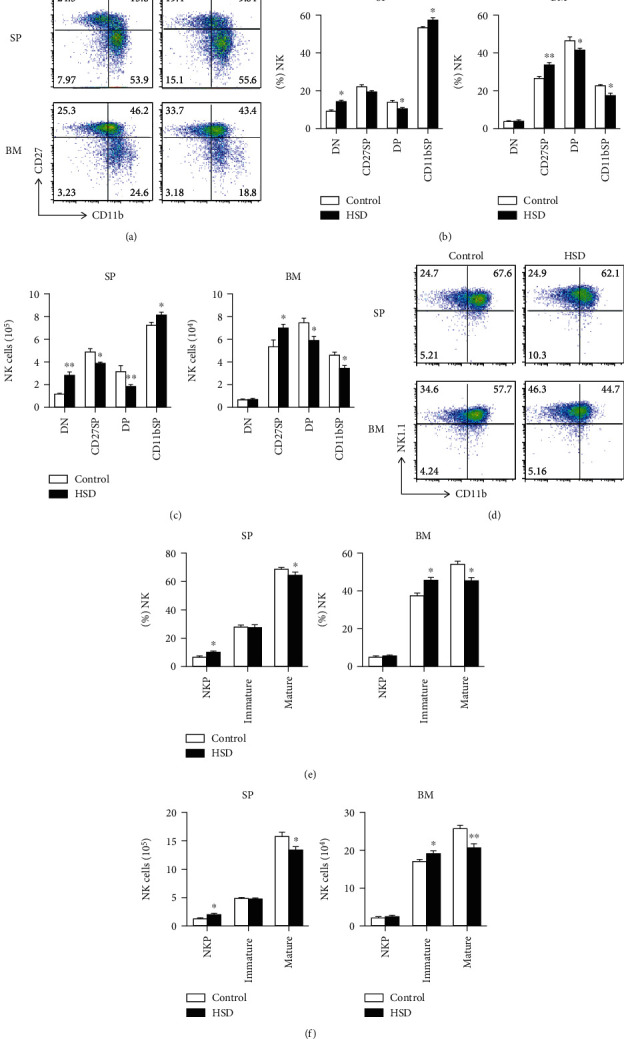
HSD reduced the number of effective NK cells in the spleen and inhibited their maturation in both the spleen and bone marrow. Representative flow cytometry profiles (a). The percentage (b) and absolute number (c) of NK cell subsets DN (CD27^−^CD11b^−^), CD27SP (CD27^+^CD11b^−^), DP (CD27^+^CD11b^+^), and CD11bSP (CD27^−^CD11b^+^) in the spleen and BM of control and HSD-fed mice. Representative flow cytometry profiles (d). The percentage (e) and absolute number of NKp (NK1.1^−^CD11b^−^), imNK (NK1.1^+^CD11b^−^), and mNK (NK1.1^+^CD11b^+^) cells gated on CD3^−^ CD122^+^ splenocytes and bone marrow cells of control and HSD-fed mice. All the data represent at least three independent experiments. Data are shown as the means ± SEM. Unpaired Student's *t*-tests (two-tailed) were performed using the Prism software. A *P* value of <0.05 was considered significant. ^∗^*P* < 0.05, ^∗∗^*P* < 0.01.

NK cells originate from hemopoietic stem cells in the bone marrow, a percentage of which remains in the bone marrow during maturation, while a peripheral portion matures in the spleen. Hence, we wondered whether HSD would differentially affect NK cell development in the spleen and bone marrow. Interestingly, the percentage and absolute number of NK precursors (CD27^−^CD11b^−^) in the spleen were increased in HSD-fed mice (Figures 3(a) and 3(b)), and the absolute number of immature NK cells (CD27^+^CD11b^−^) in the spleen was slightly decreased (Figure 3(c)). However, in the bone marrow of HSD-fed mice, the percentage and number of immature NK cells (CD27^+^CD11b^−^) were significantly enhanced, while those of mature NK cells, including CD27^+^CD11b^+^ and CD27^−^CD11b^+^, were significantly decreased, indicating that HSD inhibited NK cell maturation in the bone marrow (Figures 3(a)−3(c)). We used a further two NK markers, NK1.1 and CD11b, to distinguish between developmental stages and found that the percentage and absolute number of immature Nkp (NK1.1^−^CD11b^−^) cells were increased in the spleen of HSD-fed mice, while mature NK cells (NK1.1^+^CD11b^+^) were significantly decreased (Figures 3(d)−3(f)). In the bone marrow of HSD-fed mice, the percentage and absolute number of immature NK cells were increased, while the mature NK cells were decreased (Figures 3(d)−3(f)). These data indicate that HSD inhibits the maturation of NK cells, suggesting disruption of their function.

### 3.4. Secretion of CD107a and IFN-*γ* Is Deficient in NK Cells from HSD-Fed Mice following Stimulation with MHC-I-Deficient Tumor Cells

**Figure 4:**
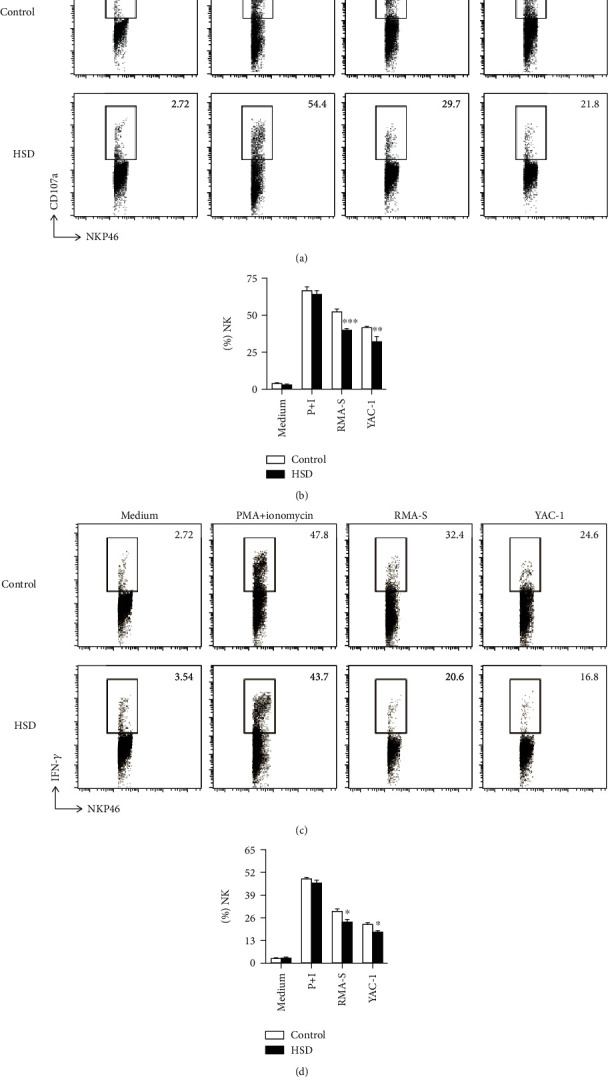
Secretion of CD107a and IFN-*γ* is deficient in NK cells from HSD-fed mice following stimulation with tumor cells. Representative flow cytometry profiles and percentage of CD107a (a, b) and IFN-*γ* (c, d) expression in poly (I:C)-activated splenic NK cells stimulated with YAC-1 or RMA-S tumor cells. All the data represent at least three independent experiments. Data are shown as the means ± SEM. Unpaired Student's *t*-tests (two-tailed) were performed using the Prism software. A *P* value of <0.05 was considered significant. ^∗^*P* < 0.05, ^∗∗^*P* < 0.01, and ^∗∗∗^*P* < 0.001.

CD107a is a glycosylated protein richly expressed on the lysosomal membrane, and its expression on the cell surface increases upon granulocyte activation [17]; thus, CD107a is a marker of NK cell degranulation. Hence, we examined the expression of CD107a in NK cells from HSD-fed mice following stimulation with target tumor cells, RMA-S or YAC-1, and found that CD107a expression was decreased by approximately 24% and 22%, respectively (Figures 4(a) and 4(b)). These data indicate that HSD impairs degranulation of NK cells. Moreover, IFN-*γ* is the main cytokine secreted by NK cells to kill tumor cells; hence, we further examined IFN-*γ* secretion by NK cells from HSD-fed mice by FACS. Splenic lymphocytes were isolated from control and HSD-fed mice and cocultured with RMA-S or YAC-1 cells to induce activation. The results show that in comparison with control mice, the secretion of IFN-*γ* in NK cells from HSD-fed mice was decreased by approximately 20% following RMA-S stimulation. Similarly, following YAC-1 stimulation, the secretion of INF-*γ* also increased by approximately 20% in the HSD-fed group (Figures 4(c) and 4(d)); therefore, the ability of NK cells from HSD-fed mice to secrete IFN-*γ* was significantly reduced. Taken together, these results indicate that the function of NK cells is abnormal following administration of HSD.

### 3.5. HSD Decreases NK Cell Proliferation but Does Not Disturb Apoptosis

**Figure 5:**
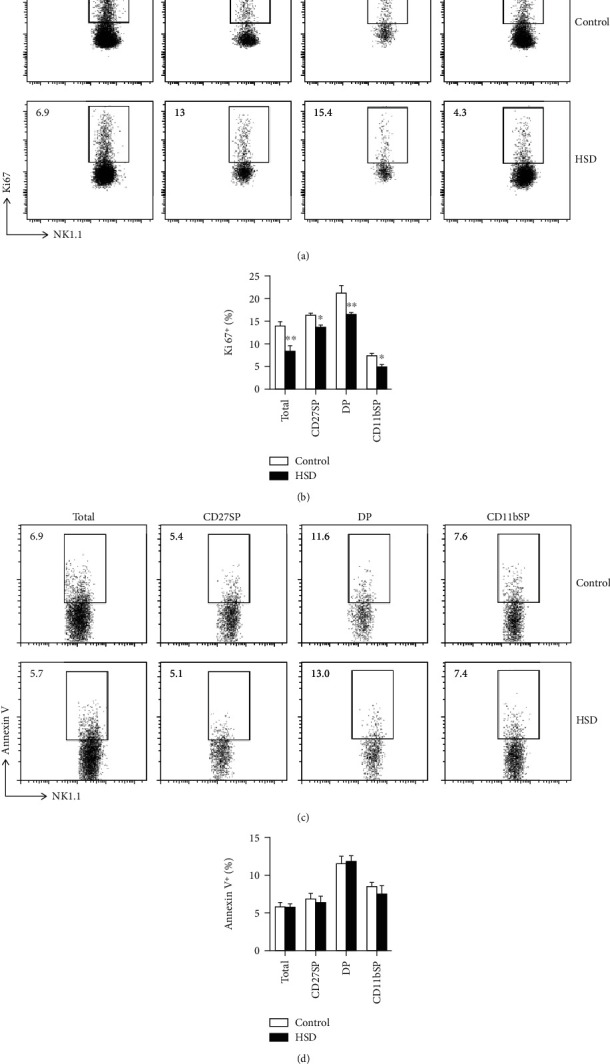
HSD decreases NK cell proliferation but does not disturb apoptosis. Representative flow cytometry profiles of Ki67^+^ splenic NK cells from control and HSD-fed mice during different developmental stages (a). The percentage of Ki67^+^ NK cells (b). Representative flow cytometry profiles of annexin V^+^ cells from control and HSD-fed mice during different developmental stages (c). The percentage of annexin V^+^ NK cells (d). All the data represent at least three independent experiments. Data are shown as the means ± SEM. Unpaired Student's *t*-tests (two-tailed) were performed using the Prism software. A *P* value of <0.05 was considered significant. ^∗^*P* < 0.05, ^∗∗^*P* < 0.01.

The number of NK cells in the spleen of HSD-fed mice was reduced; therefore, it was imperative to further explore the underlying reasons. We used flow cytometry to detect two important indicators of the maintenance of NK cells number: cell proliferation and apoptosis. Ki67 is an essential indicator of cell proliferation, and accordingly, we found that the proliferation of NK cells in the spleen of HSD-fed mice was significantly inhibited. The number of Ki67^+^ cells among the total NK cells and among NK cells of various developmental stages was significantly lower in HSD-fed mice than that in control mice (Figures 5(a) and 5(b)), indicating that the proliferation ability of NK cells was impaired in HSD-fed mice. Moreover, we also tested the apoptosis level of NK cells and found that the number of annexin V^+^ cells in the spleen of HSD-fed mice was comparable with that in the control group. There was no difference in the rate of total or annexin V^+^ NK cells at various developmental stages between control and HSD-fed mice (Figures 5(c) and 5(d)), indicating that HSD does not promote NK cell apoptosis. Based on these results, we speculate that HSD reduces the number of NK cells residing in the spleen by inhibiting proliferation.

### 3.6. HSD Decreases NK Cell Activation

**Figure 6:**
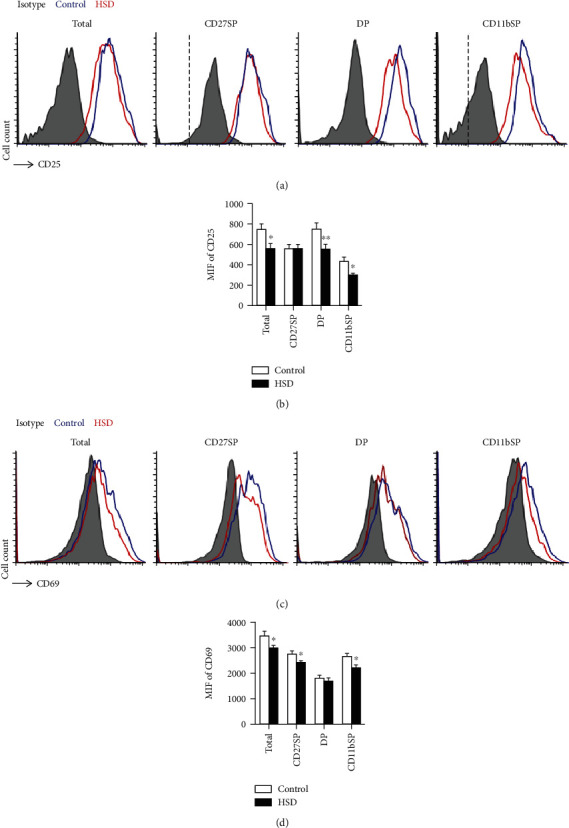
HSD decreases NK cell activation. Expression of the activation marker, CD25, in splenic NK cells from control and HSD-fed mice was assessed during different developmental stages (a). The mean fluorescence intensity of CD25 in NK cells (b). Expression of the activation marker, CD69, in splenic NK cells from control and HSD-fed mice was assessed during different developmental stages (c). The mean fluorescence intensity of CD69 in NK cells (d). All the data represent at least three independent experiments. Data are shown as the means ± SEM. Unpaired Student's *t*-tests (two-tailed) were performed using the Prism software. A *P* value of <0.05 was considered significant. ^∗^*P* < 0.05, ^∗∗^*P* < 0.01.

HSD can reduce the secretion of CD107a and IFN-*γ* by NK cells and weaken their ability to kill tumor cells; thus, it is important to further explore the mechanism by which HSD negatively affects NK cell function. We further evaluated the markers, CD25 and CD69, to determine whether HSD inhibits NK cell activation. We used flow cytometry to detect the activation of total NK cells and those at various developmental stages in the spleen. In comparison with control mice, the expression of CD25 in total NK cells in the spleen of HSD-fed mice was significantly reduced. With the exception of CD27SP NK cells, the expression of CD25 at other developmental stages was significantly reduced (Figures 6(a) and 6(b)). Moreover, we also evaluated the expression of CD69, another activation marker of NK cells, and found that its expression in total NK cells in the spleen of HSD-fed mice was significantly reduced. With the exception of CD27^+^CD11b^+^ DP NK cells, the expression of CD69 at other developmental stages was significantly reduced (Figures 6(c) and 6(d)), indicating that HSD inhibits CD69 expression in NK cells. These data show that HSD inhibits NK cell activation, which may be one of the reasons for impaired cell function.

### 3.7. HSD Increases gp91phox-Mediated ROS Production following Downregulation of CD122 in NK Cells

**Figure 7:**
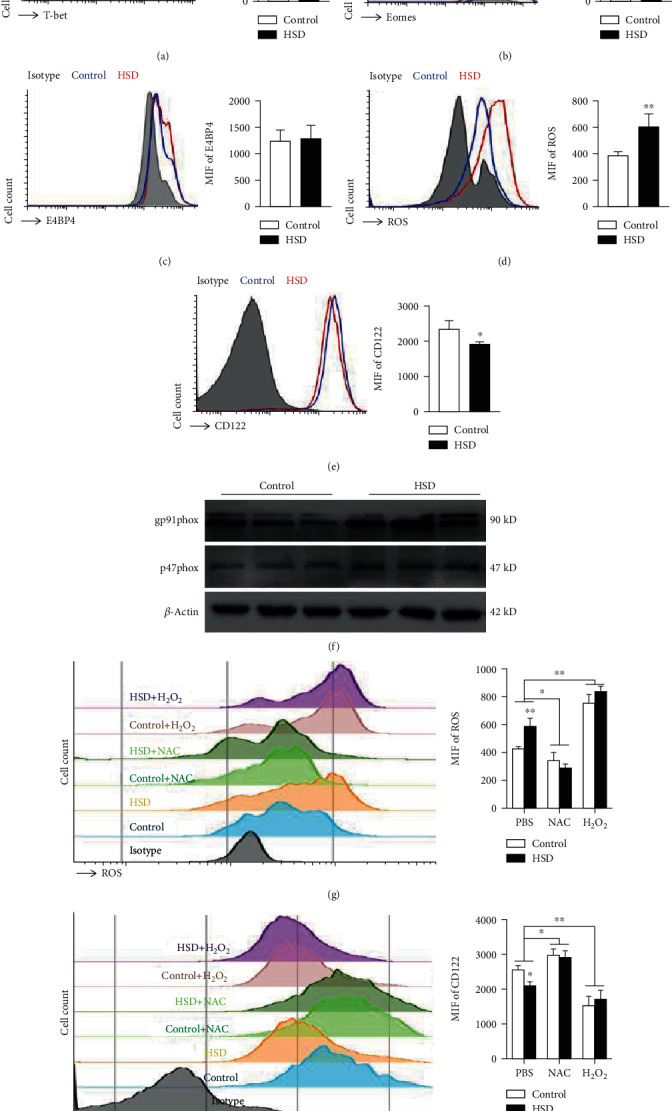
HSD increases gp91phox-mediated ROS production following downregulation of CD122 in NK cells. Representative flow cytometry profiles and mean fluorescence intensity of T-bet (a), Eomes (b), E4BP4 (c), ROS (d), and CD122 (e). Expression levels of the NADPH oxidase subunits, gp91phox and p47phox, were detected by western blotting (f). Representative flow cytometry profiles and mean fluorescence intensity of ROS in NK cells following inhibition by N-acetylcysteine or induction by H_2_O_2_ (g). Representative flow cytometry profiles and mean fluorescence intensity of CD122 in NK cells following inhibition or induction of ROS (h). All the data represent at least three independent experiments. Data are shown as the means ± SEM. Unpaired Student's *t*-tests (two-tailed) were performed using the Prism software. A *P* value of <0.05 was considered significant. ^∗^*P* < 0.05, ^∗∗^*P* < 0.01.

Finally, we explored the signaling pathway by which HSD regulates NK development and function. We used flow cytometry to detect the levels of T-bet, Eomes, and E4BP4, which are closely related to the maturation and functional acquisition of NK cells. Interestingly, the expression levels of these transcription factors that determine the fate of NK cells were unaffected in HSD-fed mice (Figures 7(a)−7(c)). Furthermore, ROS are normal products of oxygen metabolism and play an important role in cell signaling and the maintenance of homeostasis. Under stress conditions, increased ROS production can cause cellular damage; therefore, we further evaluated ROS production in NK cells. We found that ROS production in NK cells from the spleen of HSD-fed mice was significantly increased in comparison with that in control mice, indicating that the oxidative stress level of NK cells from HSD-fed mice was increased (Figure 7(d)), which will affect the expression of important signaling molecules and subsequently impact signal transduction. We further detected the expression of CD122 (IL-15 receptor *β* chain) in NK cells and found that it was significantly decreased, indicating that increased ROS in NK cells impairs the expression of CD122 (Figure 7(e)). Subsequently, we explored the underlying mechanisms of increased ROS in HSD-fed mice by detecting the expression levels of the NADPH oxidase subunits, gp91phox and p47phox, which are related to ROS synthesis. The results show that the expression of gp91phox was significantly increased in HSD-fed mice (Figure 7(f)), which may be the reason for the increased production of ROS in NK cells. To explore the relationship between ROS production and CD122 expression in NK cells, ROS scavengers and inducers were used to control ROS production. We found that ROS production in NK cells can be effectively inhibited by N-acetylcysteine (1 mM for 6 h) and effectively induced by H_2_O_2_ (1 mM for 2 h) (Figure 7(g)). The expression of CD122 was detected following inhibition or induction of ROS production in NK cells, and we found that ROS production was negatively correlated with CD122 expression in NK cells (Figure 7(h)). These results indicate that HSD induces ROS production in NK cells, consequently inhibiting CD122 expression.

## 4. Discussion

Many studies have demonstrated that HSD promotes Th-17 cell differentiation into a pathogenic form, leading to a multitude of diseases [18]. HSD significantly damages the function of mouse Treg cells [19], and NaCl increases IFN-*γ* production and promotes the occurrence of autoimmune diseases [20]. HSD also affects innate immune cells and reduces IL-4- and IL-13-induced macrophage activation [21]; however, the effect of HSD on NK cells remains unclear. Here, we found that the percentage and absolute number of NK cells in the spleen and lungs were decreased in HSD-fed mice. Moreover, the proliferation of NK cells in the spleen of HSD-fed mice was significantly inhibited, indicating that HSD reduces the number of NK cells by inhibiting their proliferation. It has been shown that the proliferation ability of NK cells declines with aging under *in vitro* culture conditions [22], suggesting that HSD may inhibit NK cell proliferation by promoting NK cell senescence.

The maturation of NK cells is key to determining their function [23]. We found that HSD reduced the number of CD27^+^CD11b^+^ NK cells in the spleen, which are known to be the most powerful subtype, the reduction of which likely affects overall NK cell function. Subsequently, we used another marker to distinguish between the developmental stages of NK cells and found that the number of mature NK1.1^+^CD11b^+^ cells was significantly reduced in the spleen and bone marrow, indicating that HSD inhibits NK cell maturation [24–26].

To verify our hypothesis, we further evaluated the ability of NK cells to secrete CD107a and INF-*γ* by isolating mouse splenic lymphocytes and coculturing them with tumor cells. We found that following stimulation with RMA-S/YAC-1, NK cells from control mice secreted a large amount of CD107a, but those from HSD-fed mice produced a significantly reduced level, indicating that HSD damages the degranulation function of NK cells. Moreover, IFN-*γ* secretion by NK cells was increased significantly in control mice following stimulation with RMA-S/YAC-1, which was much lower in HSD-fed mice. Taken together, these results indicate that HSD reduces the ability of NK cells to degranulate and secrete IFN-*γ*, reducing their antitumor function. NK cell activation can lead to the secretion of CD107a and IFN-*γ* [27]. Mature NK cells constitutively express receptors for type I interferons, namely, IL-2, IL-12, IL-15, and IL-18, which can induce their proliferation and cytokine production and augment their lytic activity [28]; thus, HSD may result in the abnormal expression of cytokine receptors, leading to impaired NK cell function.

To explore the mechanism by which HSD negatively affects NK cell function, we first detected the level of NK cell activation and found that the expression levels of CD25 and CD69 were decreased in NK cells from HSD-fed mice, indicating that HSD inhibits NK cell activation, which may be one of the reasons for the impaired NK cell function. Further, we evaluated the key transcription factors that regulate NK cell function and found that HSD does not affect the expression of T-bet, Eomes, or E4BP4. T-bet-deficient NK cells are impaired beyond the NK cell egress defect and display increased basal rates of proliferation and apoptosis [29]. Eomes-deficient mice die during early embryogenesis, indicating that Eomes is vital for development [30]. T-bet and Eomes most likely play crucial roles in regulating CD122 in NK cells, which is critical for NK cell development, and mice lacking this molecule are severely defective in peripheral NK cell number [31]. Interestingly, HSD reduces the expression of CD122, which is the *β* chain of the IL-15 receptor associated with the development and maturation of NK cells. It is speculated that HSD reduces the expression of CD122 and, consequently, the responsiveness to IL-15 signaling, thereby inhibiting NK maturation and impairing NK function. Finally, we explored the mechanism by which HSD inhibits the expression of CD122 in NK cells. ROS is produced as a byproduct of mitochondrial ATP production in the electron transport chain, but it is also produced in a regulated manner by nicotinamide adenine dinucleotide phosphate (NADPH) oxidase (NOX) and dual oxidase [32, 33], and ROS produced by NOX2 may trigger abnormal NK cell function [34, 35]. We found that HSD increased the ROS levels in NK cells, which may be caused by enhanced activity of NOX2 (gp91phox). It has been found that inhibiting FOXO1-mediated autophagy increases the level of ROS produced by NK cells, thereby inhibiting the expression of CD122 and ultimately impairing the development and function of NK cells [36]. Therefore, we speculate that HSD increases ROS production, thereby inhibiting CD122 expression, resulting in dysregulation of NK development and function.

## Figures and Tables

**Table 1:** Primer sequences used for RT-qPCR.

Name	Oligos	
GAPDH	F: CATCCACTGGTGCTGCCAAGGCTGT	R: ACAACCTGGTCCTCAGTGTAGCCCA
CD25	F: AACCATAGTACCCAGTTGTCGG	R: TCCTAAGCAACGCATATAGACCA
CD69	F: CCCTTGGGCTGTGTTAATAGTG	R: AACTTCTCGTACAAGCCTGGG
CD107a	F: CAGCACTCTTTGAGGTGAAAAAC	R: ACGATCTGAGAACCATTCGCA
IFN-*γ*	F: ATGAACGCTACACACTGCATC	R: CCATCCTTTTGCCAGTTCCTC

## Data Availability

The datasets generated for this study are available on request to the corresponding authors.
